# Short-term use of “ECMELLA” in the context of fulminant eosinophilic myocarditis with cardiogenic shock

**DOI:** 10.1186/s12872-020-01808-3

**Published:** 2020-12-10

**Authors:** Mintje Bohné, Da-Un Chung, Eike Tigges, Hendrick van der Schalk, Daniela Waddell, Niklas Schenker, Stephan Willems, Karin Klingel, Dietmar Kivelitz, Edda Bahlmann

**Affiliations:** 1 Department of Cardiology, Asklepios Clinic St. Georg, Lohmühlenstraße 5, 20099 Hamburg, Germany; 2 Department of Cardiopathology, Institute for Pathology, University Hospital Tübingen, Tübingen, Germany; 3 Department of Radiology, Asklepios Clinic St. Georg, Hamburg, Germany

**Keywords:** Eosinophilic myocarditis, Mechanical circulatory support, ECMELLA, Bridge-to-recovery

## Abstract

**Background:**

Eosinophilic myocarditis (EM) is a rare form of myocarditis. Clinical presentation is various, includes cardiogenic shock and can often be fatal. Diagnosis is based on myocardial eosinophilic infiltration in endomyocardial biopsy. Mechanical circulatory support (MCS) is often required in patients suffering from severe cardiogenic shock. Among the available MCS options the “ECMELLA” concept, a combination of left ventricular venting by Impella® device and extracorporeal life support (ECLS) is possibly able to provide the necessary time frame for diagnostics and initiation of anti-inflammatory medication in patients with fulminant myocarditis.

**Case presentation:**

We report a case of a 38‐year‐old woman who was presented to us in severe cardiogenic shock, quickly requiring hemodynamic support by an Impella CP® device. Further dramatic hemodynamic deterioration accompanied by multi-organ dysfunction required escalation of MCS via ECLS as veno‐arterial extracorporeal membrane oxygenation (VA-ECMO). After histopathological diagnosis of EM, our patient was put on immunosuppressive therapy with prednisolone. Recovery of both right and left ventricular function allowed explanation of VA-ECMO on day 4 and further hemodynamic improvement allowed removal of the Impella® device on day 9. The patient was discharged after 7 weeks with fully restored cardiac function and in a good neurological state.

**Conclusions:**

In severe cardiac shock due to fulminant EM the ECMELLA concept as bridge-to-recovery seems to be a valid option to provide the required time for diagnostics and specific therapy.

## Background

Eosinophilic myocarditis (EM) is a rare form of myocarditis that often presents fulminantly with a high mortality-rate during the acute phase [1]. EM is characterized by myocardial inflammation with eosinophilic infiltration and has been reported in association with hypersensitivity reactions [2], immune-mediated disorders such as eosinophilic granulomatosis with polyangiitis [3], parasitic infections [4], cancer [5] and pregnancy [1]. In a large series of 179 published hospitalized cases with histologically proven EM, reported in 2017 by Brambatti et al., 35% remained with unknown underlying causes, thus classified as idiopathic or undefined [1]. Diagnosis is based on myocardial eosinophilic infiltration in endomyocardial biopsy [1, 6] and is often discovered only on postmortem examination [7]. In-hospital mortality due to heart failure is reported in 22.3%, increasing to 36.1% in hypersensitivity EM, the most frequent condition [1]. Particularly, fulminant EM with concomitant cardiogenic shock is associated with high in-hospital mortality [8] and, besides inotropic support, may require mechanical circulatory support (MCS), such as extracorporeal life support (ECLS) as a bridge to myocardial recovery [1, 9–12]. Veno-arterial extracorporeal membrane oxygenation (VA-ECMO) is an established ECLS in severe cardiogenic shock [13–15]. However, increase in systemic afterload and left ventricular (LV) end diastolic pressure are hemodynamic consequences inherent to this type of MCS, potentially impeding myocardial recovery. A combination with antegrade MCS via the antegrade LV-to-aorta Impella® microaxial device (Abiomed. Danvers, MA, USA) for LV-unloading has been described as beneficial when compared to VA-ECMO alone [16, 17]. Combining both, Impella® and ECLS is called “ECMELLA” or “ECPELLA” concept [10]. To the best of our knowledge, we present the first case of a fulminate cardiogenic shock due to EM successfully using the “ECMELLA” approach as a bridge to recovery.

## Case presentation

A 38-year-old woman presented to our emergency department with intermitting fever for the past 4 weeks, accompanied by unproductive cough, headache and dizziness, which she self-medicated with acetaminophen and metamizole. On the day of admission, she also reported nausea and abdominal pain. Seven months ago, she had had an uncomplicated delivery of a healthy child, preceded by an uneventful pregnancy. She had a medical history of radiochemotherapy for Hodgkin's disease in 2004 and had been diagnosed with ulcerative colitis in 1998, currently being medicated with mesalazine. A previous allergic reaction to penicillin had been described. Preclinical serology and laboratory results revealed DNA sequences for cytomegalovirus, a slight increase of the inflammatory marker serum C-reactive protein (41.6 mg/l) and a normal procalcitonin serum value.

**Fig. 1:**
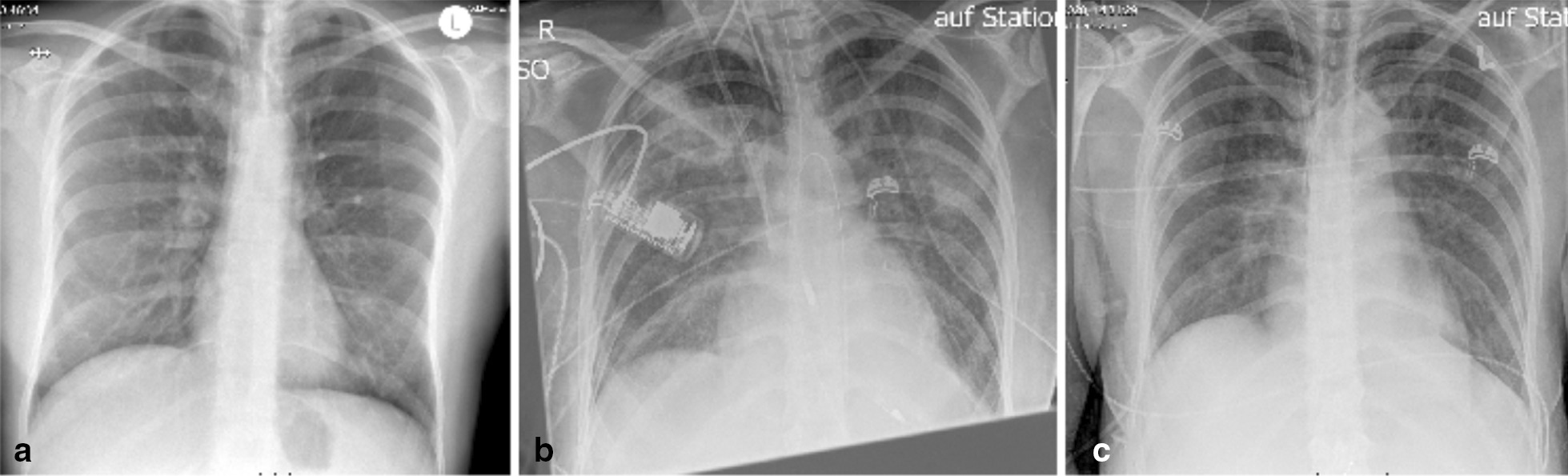
Chest radiography in supine position and anterior–posterior projection at admission (**a**), after Impella® placement (**b**) and at discharge (**c**). **a** Marked pulmonary congestion and mild bilateral pleural effusion. **b** Prominent bilateral pulmonary edema. **c** Residual pulmonary congestion

**Fig. 2:**
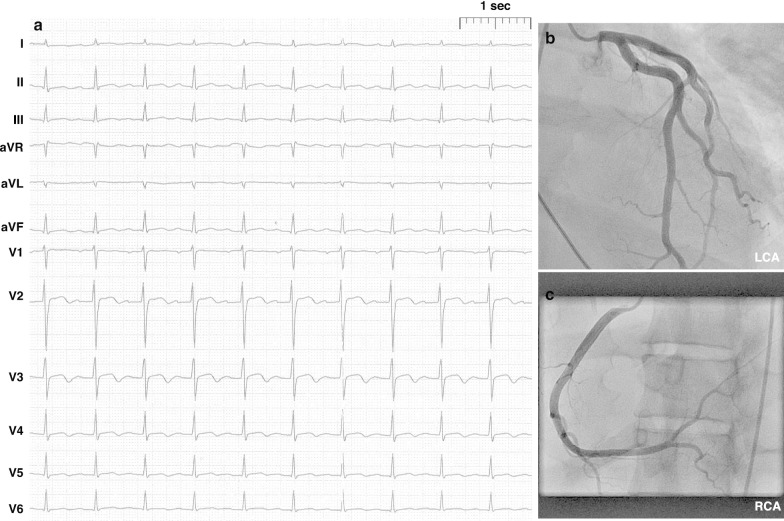
**a** 12-lead electrocardiogram (25 mm/s) 2 weeks after admission showing sinus rhythm (90 bpm), narrow QRS complexes and T-wave inversions in lead V3–V6. **b**, **c** A coronary angiogram of the left (LCA) and right (RCA) coronary artery with regular vessel anatomy without signs of coronary artery disease

**Fig. 3:**
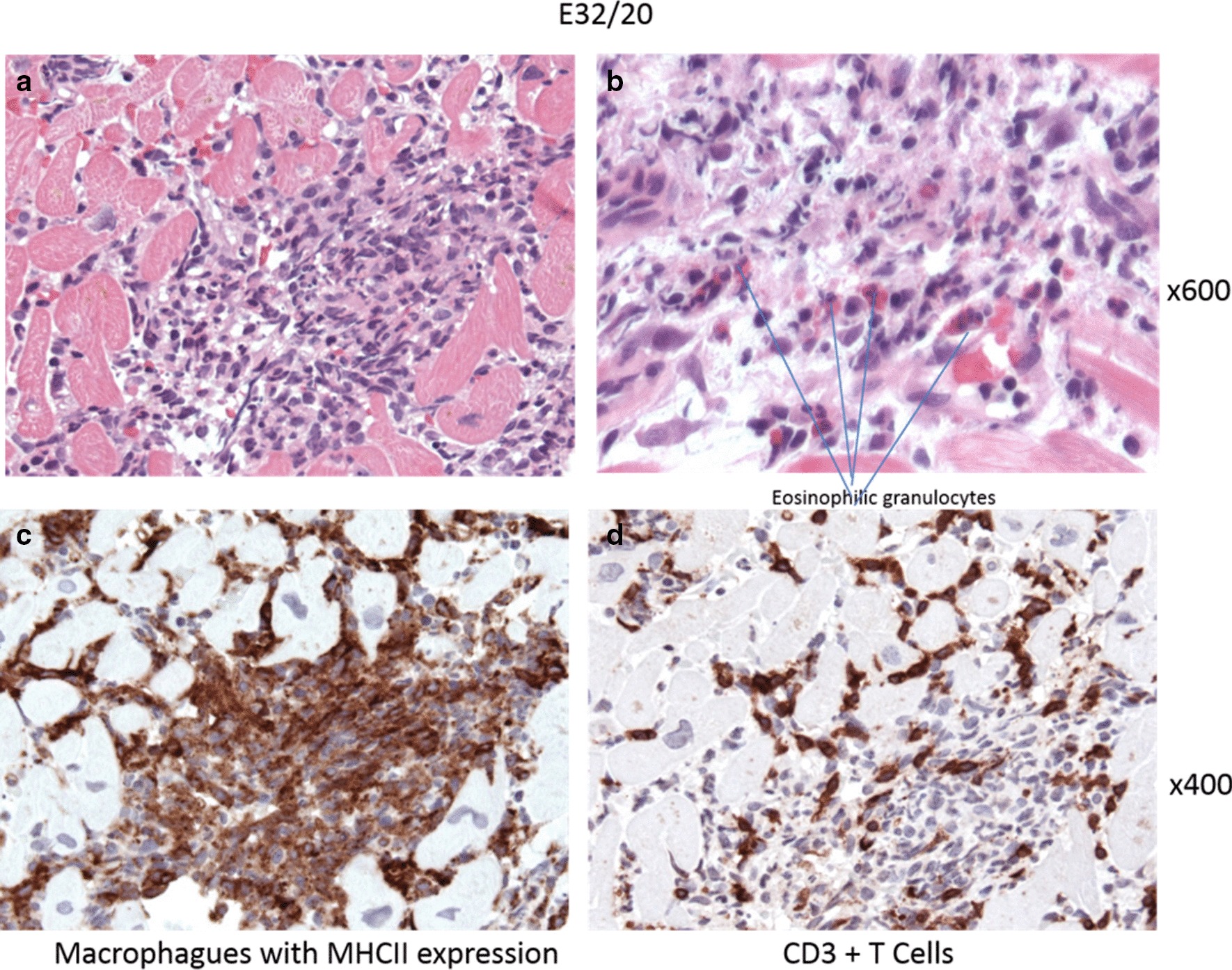
Histology of the LV endomyocardial biopsies (hematoxylin–eosin staining) demonstrating severe acute eosinophilic myocarditis (**a**). **b** Myocardial infiltration with eosinophilic granulocytes in an enlarged section of (**a**). **c** Immunohistochemical staining for MHC II (mainly infiltrates of macrophages) and **d** CD3 + T cells

**Fig. 4:**
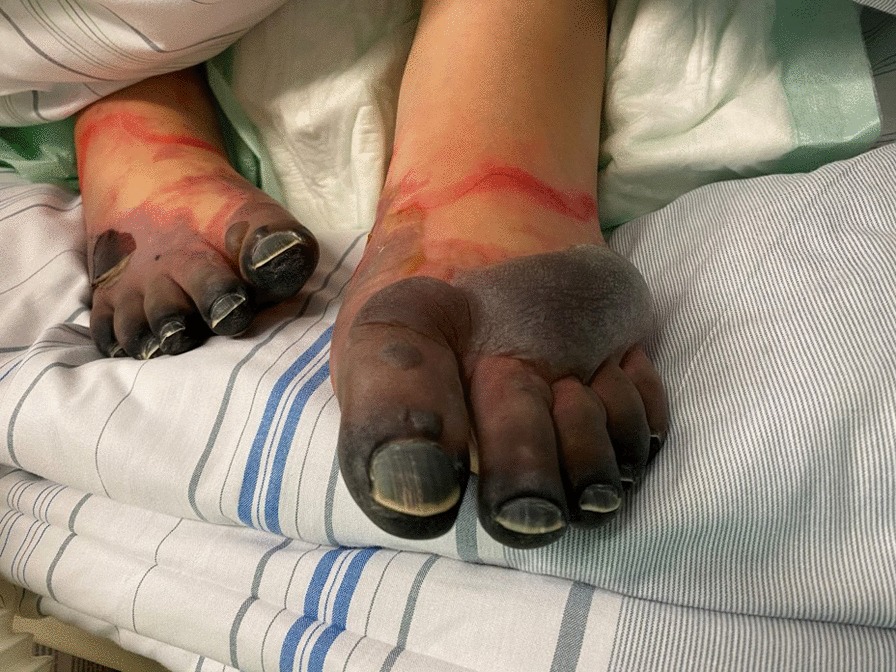
Photograph of bilateral pedal necrosis from the tips of all toes to the distal metatarsal section

**Fig. 5:**
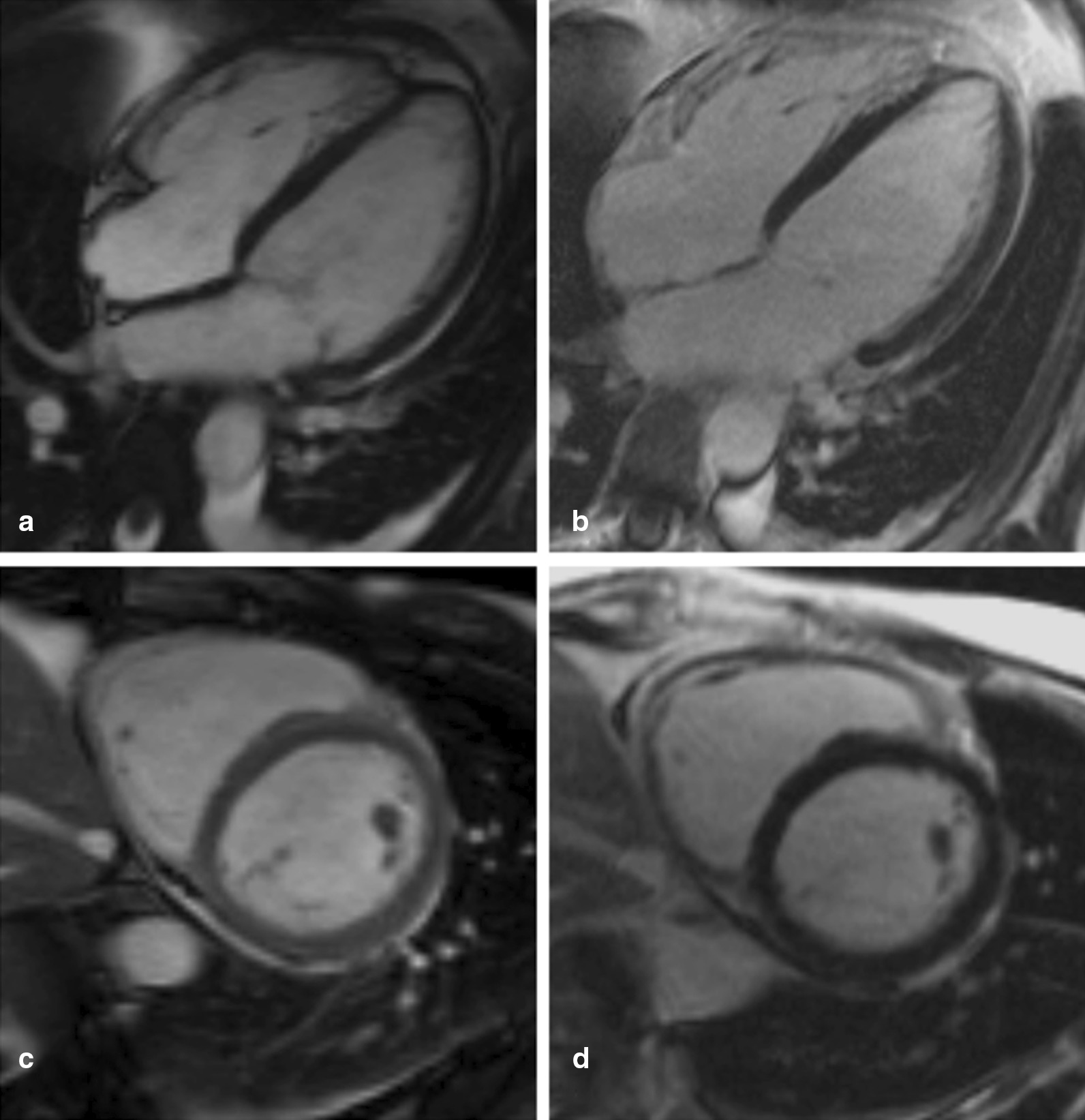
Cardiac MRI 3 weeks after admission shows 4 chamber views (**a**, **b**) and short axis views (**c**, **d**) in a functional test (cine MRI, **a**, **c**) and late enhancement (**b**, **d**), respectively, mildly enlarged systolic and diastolic volumes, a borderline reduced systolic LV function without myocardial late gadolinium enhancement (LV ejection fraction 56%, end-diastolic volume 87 ml/m^2^, end-systolic volume 38 ml/m^2^, stroke volume 48 ml/m^2^). Cardiac MRI images showed a minimal percicardial effusion and no evidence of myocardial oedema

The patient showed rapid deterioration of her condition and was transferred to the intensive care unit. She had a body temperature of 38.6° Celsius, a heart rate of 140 beats/min, a blood pressure of 80/40 mmHg, a respiratory rate of 30/min and suffered of tarnished consciousness. The electrocardiogram revealed sinus tachycardia and nonspecific ST-T changes. Chest radiography showed prominent pulmonary edema and bilateral pleural effusion (Fig. 1a). Transthoracic echocardiography (TTE) showed highly impaired left ventricular (LV) ejection fraction of < 30%, slight pericardial effusion and no intraventricular thrombus. Laboratory results revealed increased markers of cardiac injury and congestion (high-sensitive Troponin I 3323 ng/l, N-terminal pro brain natriuretic peptide 21053 ng/l) and inflammatory response (C-reactive protein 229 mg/l, white blood cell count of 29.3/ nl with 82% neutrophils), impaired renal function (blood urea nitrogen 56 mg/dl and creatinine 1.2 mg/dl), mild elevation of transaminases (aspartate aminotransferase 289 U/l and alanine aminotransferase 71 U/l). Empirical broad-spectrum antibiotics and specific antiviral therapy with meropenem, clarithromycin, gentamicin and valganciclovir was established. Chest computed tomography (CT)-scan confirmed prominent pulmonary edema, accumulation of pleural effusion and infiltrates indicating possible pneumonia. There was no evidence of any other infectious focus in the abdominal CT-scan, neurological exanimation including lumbar puncture and gynecological examination. Follow-up TTE showed further decline of left ventricular ejection fraction, combined with rising vasopressor demand, rapidly increasing laboratory signs of cardial decompensation (maximal N-terminal pro brain natriuretic peptide > 70,000 ng/l, lactate dehydrogenase 13,000 U/l) and positive evidence for disseminated intravascular coagulation (thrombocytopenia of 95/nl, prolongation of partial thromboplastin time of 56 s, decreased antithrombin III). An Impella CP® device was inserted on day 1 shortly after her initial presentation (Fig. 1b), followed by a LV endomyocardial biopsy. Urgent coronary angiography in a patient with cardiogenic shock showed no underlying coronary heart disease (Fig. 2). After initiation of the Impella CP®, the patient displayed further respiratory decline, requiring mechanical ventilation. In light of rising serum lactate levels (maximum 16.0 mmol/l), rapidly increasing catecholamine demand and additional imposing right ventricular failure, MCS was escalated via VA-ECMO at the same day. Additionally, the patient was started on inodilatative therapy with levosimendan. Anuria and acute renal failure required renal replacement therapy. Extracorporeal cytokine hemoadsorption via CytoSorb® was installed for 48 h [17]. Histology of the LV endomyocardial biopsies revealed severe acute EM showing myocardial infiltration with eosinophilic granulocytes, macrophages and CD3 + T cells (Fig. 3). Further laboratory work up revealed no peripheral eosinophilia and increased levels of cytomegalovirus-DNA (437 IE/ml). Perinuclear anti-neutrophil cytoplasmic antibodies (p-ANCA) and cytoplasmic anti-neutrophil cytoplasmic antibodies (c-ANCA) were negative. Immunosuppressive therapy with prednisolone was initiated on day 7 (1 mg/kg/day) and continued for 14 days, followed by a dose tapering regimen of 10 mg every 4 weeks. On day 5 both feet showed signs of acute gangrenous necrosis from the tips of all toes to the metatarsales (Fig. 4). Duplex sonography revealed normal flow patterns in all lower extremity arteries and ruled out arterial flow obstruction by femoral ECMO cannulas and Impella® introducer sheath. The level of inotropic support required to maintain stable hemodynamics during “ECMELLA” use constantly decreased. VA-ECMO could be removed after 4 days. LV function recovered quickly to an ejection fraction of 55% by day 6, while the patient was still on mechanical circulation Impella® device support and without inotropes. The Impella® device was gradually weaned and could be removed after 9 days. The patient was extubated on day 13, had to be re-intubated due to palate bleeding the same day and could be successfully extubated the following day. Oral heart failure medication (e.g. angiotensin-converting-enzyme inhibitor and *β*-blocking agent) was established after ongoing hemodynamic stability. Follow-up chest radiography showed a normalized cardiac silhouette and only residual mild pulmonary edema (Fig. 1c). Cardiac magnetic resonance imaging (MRI) after 28 days demonstrated a borderline reduced systolic LV- and intact right ventricular function without signs of inflammatory myocardial changes or endomyocardial fibrosis (Fig. 5). During the whole course of hospitalization our patient was in sinus rhythm and did not present any arrhythmic events. The patient was discharged from intensive care unit after 6 weeks in good clinical condition without neurological deficit and renal recovery and was transferred to a rehabilitation center thereafter. In consequent ambulatory follow-up visits, normal parameters were obtained for cardiac function. Six month later, transmetatarsale amputation of the right digiti III-V and resection of the distal phalanxes of the right digitus II and left digiti III-V became necessary.

## Discussion and conclusions

Due to the severity of symptoms and extend of end-organ damage seen in this patient, we considered this a case of fulminant myocarditis (FM), characterized by an acute myocardial inflammation leading to cardiogenic shock [10, 11, 18]. As shown in a large registry of 220 patients with histologically proven acute myocarditis, patients with FM have higher rates of cardiac death and heart transplantation compared to non-fulminant myocarditis with 28% vs. 1.8% [18]. Stages in EM include myocardial infiltration of eosinophils causing acute necrosis, followed by hypercoagulation, potentially leading to thrombosis, either within the coronary vasculature or the ventricles in up to 13.7% [1], which can ultimately result in permanent cardiac dysfunction due to myocardial scar formation [18]. Clinical presentation in the often young patients [1] can vary, including embolic cerebral infarctions [19]. After excluding potential embolic sources, vasoconstrictive hypoxia due to high catecholamine demand and disseminated intravascular coagulation was assumed as the most likely cause for the pedal necrosis due to end-organ hypoperfusion in our patient [20]. Moreover, thrombosis is known to complicate MCS [21]. The underlying cause of EM in our patient remained uncertain. Pre-existing diseases in the patient`s history as Hodgkin's disease and ulcerative colitis and the recent pregnancy, however are described as possible associated conditions [1]. Prior to the histological proof of EM, a systemic cytomegalovirus reactivation was also considered as the underlying cause [22]. An important differential diagnosis includes drug reaction (even in absence of detectable peripheral eosinophilia) [23, 24]. In our case, specific information on the timings of any medication use prior to hospitalisation was positive for mesalazine only. Features that point against eosinophilic granulomatosis with polyangiitis as the diagnosis were missing specifities as asthma and blood eosinophilia and ear, nose and throat involvement, although it is known that ANCA is only positive in a proportion of eosinophilic granulomatosis patients [25]. Cardiac MRI showing no significant fibrosis suggests an acute aetiology. Peripheral eosinophilia, as in our case, is absent in up to 25% of patients with EM [1] and underscores the importance of endomyocardial biopsy in cases of unexplained abrupt LV impairment [26, 27]. Immunosuppression is the most common treatment modality for EM [1], although no clinical trial has tested its efficacy. Remarkable is the observation that the patient started to improve prior to initiation of immunosuppression with a rapid restitution of the biventricular function. Thus, considering also the positivity for cytomegalovirus, the initiated steroid therapy was probably not necessary in our case. The extent of profound cardiac- and multiorgan dysfunction in our patient required immediate MCS allowing time to initiate positive inotropic pharmacotherapy, recommended for the management of patients with acute heart failure to help myocardial recovery and for making the proper diagnosis [12, 13]. For patients in FM, ECLS via VA-ECMO is the most commonly described MCS [9, 11, 15]. However, it should be taken into account that VA-ECMO increases LV afterload severely, resulting in LV distention and pulmonary edema. The Impella® device provides circulatory support with LV-venting, reduces LV-afterload [10] and has been described as sole therapy in rare cases of acute FM [28, 29]. However, in patients with concomitant right ventricular dysfunction, the Impella is ineffective and can be even counterproductive by exposing the patient to the adverse effects of such support (anemia, complications related to vascular access, distal limb ischemia, infections) [21]. In this specific clinical context, VA-ECMO may be considered as first MCS alternative and, eventually, Impella as LV venting device. In our patient, due to the rapidly developing also right ventricular dysfunction, the Impella® device could not provide adequate hemodynamic support. The MCS strategy was therefore escalated to the ECMELLA concept as the next reasonable step. After additional implantation of a VA-ECMO, the hemodynamic situation gradually stabilized. Continued circulatory support with LV-venting and inodilatative therapy allowed quick recovery of LV function.

Even in fulminant cardiogenic shock due to EM and consecutive biventricular heart failure with multi-organ dysfunction, the ECMELLA concept can be an effective way of MCS therapy providing the time frame for decision making processes, initiation of specific therapy in terms of inotropic and immunosuppression support and to allow cardiac reconstitution. This adds to current evidence, however further research is needed to verify the ECMELLA concept in a wider clinical use.

## Data Availability

Please contact author for data requests.

## Abbreviations

CT: Computed tomography
ECLS: Extracorporeal life support
EM: Eosinophilic myocarditis
FM: Fulminant myocarditis
LV: Left ventricle
MCS: Mechanical circulatory support
MRI: Cardiac magnetic resonance imaging
TTE: Transthoracic echocardiography
VA-ECMO: Veno-arterial membrane oxygenation
